# Draft genome sequences of two *Thioalkalivibrio sulfidiphilus* strains isolated from a pilot-scale haloalkaline biodesulfurization installation

**DOI:** 10.1128/mra.00475-25

**Published:** 2025-09-24

**Authors:** Caroline M. Plugge, Suyash Gupta, Sarahi Garcia Villa, Peer H. A. Timmers, Gerard Muyzer

**Affiliations:** 1 Wetsus, European Centre of Excellence for Sustainable Water Technology 361182, Leeuwarden, the Netherlands; 2 Laboratory of Microbiology, Wageningen University and Research, Wageningen, the Netherlands; 3 Microbial Systems Ecology, Department of Freshwater and Marine Ecology, Institute for Biodiversity and Ecosystem Dynamics, University of Amsterdam 1234, Amsterdam, the Netherlands; 4 Department of Environmental Sciences for Sustainability, IE University 378523, Segovia, Spain; Portland State University, Portland, Oregon, USA

**Keywords:** *Thioalkalivibrio*, sulfide, biodesulfurization

## Abstract

The genome sequences of two *Thioalkalivibrio sulfidiphilus* strains, SGAR-7 and SGAR-13, isolated from a pilot-scale haloalkaline biodesulfurization (BD) installation, are reported. The genomes comprise Flavocytochrome c sulfide dehydrogenase and SoxAX cytochromes, which are essential for respiration and thiosulfate oxidation, offering insights into *Thioalkalivibrio*’s role in BD.

## ANNOUNCEMENT

Although numerous strains have been isolated from natural sources, *Thioalkalivibrio* species have also been identified as key players in man-made BD systems, where they convert sulfide to elemental sulfur (1–5). We enriched and isolated the most dominant sulfide and thiosulfate-oxidizing bacteria from a pilot-scale haloalkaline BD system through serial dilution. Two isolates, *Thioalkalivibrio sulfidiphilus* strains SGAR-7 and SGAR-13, were obtained, and their genomes were sequenced. Default parameters were used for all analyzes except where otherwise noted.

SGAR-7 and SGAR-13 were enriched and routinely grown in serial dilution under oxygen-limiting conditions (12% vol/vol) in a haloalkaline mineral medium at pH 8.5 in serum bottles at 35°C. The medium was prepared as described previously (6), with 20 mM sodium thiosulfate and without urea. The highest dilutions with growth were streak plated on the same medium (6) with 2% (wt/vol) washed agar. SGAR-7 and SGAR-13 were obtained in pure culture, phylogenetically analyzed by sequencing the 16S rRNA gene (7) and genome sequencing. The taxonomic identity of both strains was confirmed using the GTDB-Tk classifier (8). Genomic DNA was extracted using the modified CTAB method (9,10) and sequenced with the Illumina NovaSeq 6000 and Oxford Nanopore sequencing platforms. For Illumina, the DNA concentration, purity, and integrity were analyzed using the Agilent 5400 fragment analyzer. For library preparation, the DNA was randomly sheared into short fragments using a standard kit for NovaSeq 6000. Fragments were repaired, A-tailed, and ligated with Illumina adapters. Adapter-containing fragments were amplified by PCR, size-selected for 350 bp, and purified. Libraries were checked using Qubit and real-time qPCR and pooled. Paired-end 150 bp sequencing was done with the Illumina NovoSeq 6000 sequencer. DNA quality checks, library preparation, and sequencing were performed at Novogene Ltd., Cambridge, UK.

Raw sequencing data were cleaned of adapter sequences, reads with *N* > 10%, and low-quality reads (*Q*-score <5).

The DNA library for Oxford Nanopore sequencing was prepared using Nanopore Kit SQK-LSK109 (Oxford Nanopore, UK) without DNA shearing. Sequencing was performed on the MinION platform using a FLO-FLG001 flow cell with 9.4.1 chemistry, followed by base calling with the MinKNOW v21.02.2 and Guppy v4.3.4 protocol. Raw read quality control was conducted using MinKNOW.

Hybrid genome assembly was performed using Unicycler v0.4.9 under standard conditions for both strains (11). For SGAR-7, a complete circular genome was generated, measuring 3,277,547 bp. SGAR-13 was assembled into three contigs (3,270,812, 41,633, and 377 bp). The quality of the assembled genomes was assessed with CheckM v1.4.0 (12). Subsequently, the assembled genomes were annotated using the NCBI with the NCBI Prokaryotic Genome Annotation Pipeline (13). Further genome characteristics are shown in Table 1.

**TABLE 1:** Genome statistics of *Thioalkalivibrio sulfidiphilus* strain SGAR-7 and SGAR-13

	Strain SGAR-7	Strain SGAR-13
BioSample accession no.	SAMN47214803	SAMN47214838
Raw reads Illumina	8,190,030	8,713,036
Raw reads Nanopore	39,340	77,656
Genome size (bp)	3,277,547	3,312,445
Genome coverage Illumina (×)	2.5	2.6
Genome coverage Nanopore (×)	1.9	2.6
Number of contigs	1 (circular)	3
*N*_50_ value Nanopore (Kb)	14.85	13.54
CheckM completness (%)	99.94	99.94
CheckM contamination (%)	0.75	1.72
DNA coding region (CDS)	3,123	3,166
G + C content (%)	65.32	65.38
Total genes	3,175	3,218
RNA genes	52	52
rRNA operons (5S, 16S, 23S)	1, 1, 1	1, 1, 1
Protein-coding genes	3,075	3,122
Pseudo genes	48	44

## Data Availability

Draft genome sequences were deposited at DDBJ/ENA/GenBank via BioProject PRJNA1231550. All sequence reads are available from the NCBI Sequence Read Archive under accession number SRP570516. The genome accession for SGAR-7 is CP183315 at DDBJ/ENA/GenBank, and the whole-genome shotgun project for SGAR-13 is available under JBLZB01000000.
